# Prognostic Value of the PET/CT-Derived Maximum Standardized Uptake Value Combined with the Neutrophil–Lymphocyte Ratio in Patients with Hepatocellular Carcinoma Undergoing Hepatectomy

**DOI:** 10.3390/curroncol33010013

**Published:** 2025-12-25

**Authors:** Tianyi Zhou, Chaoliu Dai

**Affiliations:** Department of General Surgery, Shengjing Hospital of China Medical University, Shenyang 110004, China; zhouty@sj-hospital.org

**Keywords:** hepatocellular carcinoma, surgical resection, PET/CT SUVmax, neutrophil–lymphocyte ratio, overall survival, disease-free survival

## Abstract

**Background:** We aimed to evaluate ability of a novel scoring system that combines fluorodeoxyglucose-uptake parameters and systemic inflammatory response indicators to predict hepatocellular carcinoma (HCC) prognosis. **Methods:** Clinical data were collected from patients with HCC who underwent hepatectomy at our hospital in 2014–2022. The tumor-to-liver ratio (TLR) was adopted as a positron emission tomography/computed tomography (PET/CT) standardized uptake value (SUV)-related indicator and calculated as the ratio of the SUV_max_ of tumor tissue to the SUV_mean_ of normal liver tissue. The patients’ immune microenvironment reflected the NLR. Postoperative overall survival (OS)- and disease-free survival (DFS)-related independent prognostic factors were analyzed using Cox proportional hazards regression modeling. **Results:** Eighty-nine patients were included. TLR, NLR, and alpha-fetoprotein levels were independently associated with OS and DFS. The OS and DFS in the zero-point group were significantly longer than those in the one- and two-point groups. Time-dependent ROC curve analyses revealed area under the curve values of 0.830 and 0.752 for 5-year OS and DFS, respectively, for the scoring system, outperforming single evaluation indices. **Conclusions:** The proposed scoring system, which incorporates both TLR and NLR, simultaneously reflects metabolic tumor characteristics and the host’s immune microenvironment, enabling more accurate patients with early to intermediate-stage HCC undergoing hepatectomy classification and better prognostic evaluation.

## Simple Summary

Despite recent advances, survival outcomes in patients with hepatocellular carcinoma remain poor and recurrence rates are high. Positron emission tomography/computed tomography plays an important role in the prognostic assessment of patients with hepatocellular carcinoma, as it can provide information about the morphology of metastatic lesions and the metabolic characteristics of the tumor itself. The neutrophil–lymphocyte ratio is an indicator of both the pro-tumor inflammatory microenvironment and the host’s immune status in hepatocellular carcinoma. However, it is often impossible to accurately assess the prognosis of such patients based on a single indicator alone. In this study, we established a new scoring system incorporating both preoperative positron emission tomography/computed tomography-derived standardized uptake values and the neutrophil–lymphocyte ratio. It was capable of accurately identifying those with a high recurrence risk or worse overall survival outcome, which could be useful for facilitating the selection of treatment regimens before surgery.

## 1. Introduction

Hepatocellular carcinoma (HCC) is the sixth most prevalent type of malignant tumor globally and the third leading cause of cancer-related mortality, with both the incidence and mortality rates of the disease exhibiting annual upward trends [1,2]. The burden of hepatitis is particularly high in Asia, with the region accounting for approximately 70% of all HCC cases reported worldwide [3]. Currently, the two primary radical treatment options are hepatectomy and liver transplantation. While some studies have reported enhanced survival rates for alternative therapies such as radiofrequency ablation, transarterial chemoembolization, and systemic therapy [4,5,6], no evidence exists to suggest that any of these treatments surpass surgical interventions in terms of efficacy. Therefore, surgery remains the gold standard for HCC management. Nevertheless, compared to that of other malignancies, HCC exhibits a notably high 5-year recurrence rate of 50–70%, resulting in an actual 5-year survival rate of only 18–22% [7]. This recurrence, coupled with the disease’s poor prognosis, poses a significant challenge for hepatobiliary surgeons; thus, the ability to accurately predict the prognosis of a patient prior to surgery is crucial for devising the optimal comprehensive treatment strategy. In China, HCC is strongly associated with hepatitis, and accumulating evidence suggests that tumor-associated inflammatory responses can facilitate tumor progression through various mechanisms, such as angiogenesis, invasion, and metastasis; these processes are associated with poor prognoses in patients with tumors [8,9,10,11,12,13], with the neutrophil–lymphocyte ratio (NLR) serving as an indicator of both the pro-tumor inflammatory microenvironment and the host’s immune status. Research has demonstrated that an elevated preoperative NLR is correlated with adverse outcomes in patients with HCC, and modulating the ratio may represent a potential therapeutic strategy for future HCC interventions [14].

In recent years, the use of positron emission tomography (PET)/computed tomography (CT) for preoperative examinations has become increasingly common in clinical practice. Although this imaging technique cannot effectively diagnose HCC, particularly well-differentiated HCC, it continues to play an important role in the prognostic assessment of patients with the disease, as it can provide information about the morphology of metastatic lesions and the metabolic characteristics of the tumor itself. However, it is often impossible to accurately assess the prognosis of patients with HCC based on a single indicator alone. Some studies have investigated the combined use of tumor-related inflammatory response indicators and PET/CT standardized uptake value (SUV)-related parameters to evaluate the prognosis of patients with various cancers, including non-small cell lung cancer and pancreatic cancer [15,16], the results of which have been very good; however, none have evaluated such an approach for predicting patient prognosis in HCC. Therefore, the aim of this study was to evaluate the ability of a novel scoring system to accurately predict the prognosis of patients with HCC based on a combination of fluorodeoxyglucose (FDG) uptake parameters and systemic inflammatory response indicators.

## 2. Materials and Methods

### 2.1. Case Data and Inclusion/Exclusion Criteria

**Figure 1:**
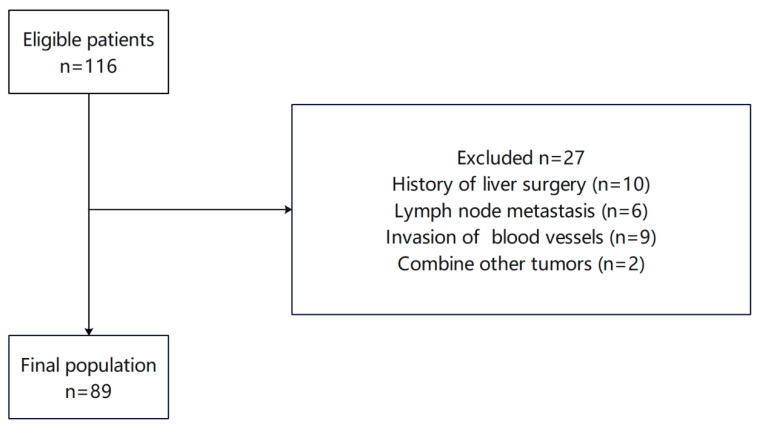
Flowchart of the cohort selection process.

Data were collected retrospectively from patients treated between 2014 and 2022 at Shengjing Hospital of China Medical University. All participants had received PET/CT scans and subsequent hepatectomy and were postoperatively diagnosed with HCC. Eligibility for the study required fulfillment of the following inclusion criteria. (1) preoperative PET/CT examinations conducted to rule out lymph node and distant metastases; (2) preoperative Child-Pugh Class A/B; (3) performance of hepatectomy; and (4) postoperative pathology-confirmed HCC with negative margins. The exclusion criteria were as follows: (1) failure to undergo splenectomy before hepatectomy; (2) the presence of other concurrent malignancies before or after surgery; (3) a diagnosis of secondary liver malignancy; (4) the occurrence of preoperative systemic infection or other viral infections; (5) presurgical administration of pharmacotherapies that could affect blood cell counts; (6) death during the perioperative period; and (7) missing SUV-related data. Ultimately, 89 patients satisfied the criteria and were included in the analysis (Figure 1). The operation times for each patient were as follows: 1 case in 2014, 5 cases in 2015, 11 cases in 2016, 8 cases in 2017, 7 cases in 2018, 9 cases in 2019, 14 cases in 2020, 14 cases in 2021, and 20 cases in 2022. This study was approved by the Ethics Committee of Shengjing Hospital (approval code: 2023PS699K). According to national legislation and institutional requirements, written informed consent for participation was not required for this study.

### 2.2. PET/CT and Research Indicators

**Figure 2:**
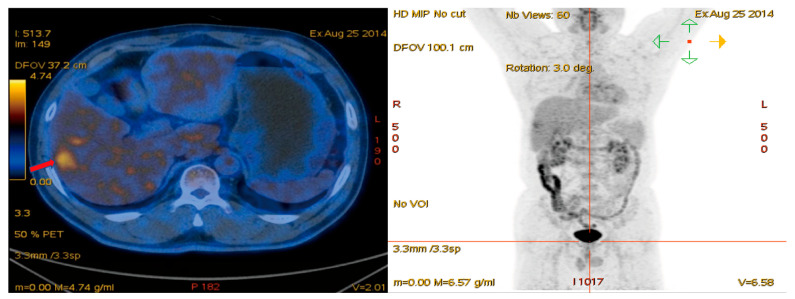
Representative imaging and the calculation of parameters in a patient with HCC. In this example, the TSUV_max_ is 4.13, the LSUV_mean_ is 2.3, and the TLR is 1.80. HCC, hepatocellular carcinoma; LSUV_mean_, average standardized uptake value in normal liver tissue; TLR, tumor-to-liver ratio; TSUV_max_, maximum standardized uptake value in tumor tissue.

PET/CT was conducted using a GE Discovery Elite PET/CT scanner (GE Healthcare, Waukesha, WI, USA). Patients were required to fast for 6 h before the examination. ^18^F-FDG was injected based on each patient’s blood glucose levels. After 1 h, the scan was performed, and the PET images were reconstructed. Two experienced nuclear medicine physicians diagnosed each case based on CT imaging in combination with the selection of regions of interest based on ^18^F-FDG concentrations. The SUV is a semi-quantitative indicator, and the maximum SUV of the tumor tissue (TSUV_max_) was calculated directly. Three regions of interest were delineated in the left and right lobes of the normal liver, and the mean SUV of normal liver tissue (LSUV_mean_) was calculated as the average of the measurements in those three regions. The tumor-to-liver ratio (TLR) was calculated as the ratio of TSUV_max_ to LSUV_mean_. Representative imaging is shown in Figure 2.

The NLR was determined based on cell counts quantified from fasting venous blood samples collected in the morning hours within the one-week period before surgery. The albumin–bilirubin (ALBI) grade was calculated by the nomogram as follows:

(log_10_ bilirubin (in μmol/L) × 0.66) + (albumin (in g/L) × −0.085).

ALBI grades of 1, 2, and 3 corresponded to scores of ≤−2.60, ≤−2.60 to ≤−1.39, and >−1.39, respectively.

The diagnosis criteria for MASLD in this study are as follows: 1. Imaging findings of liver steatosis. 2. Presence of at least one metabolic risk factor. 3. Excluding other causes of liver injury, such as active viral hepatitis.

Data were recorded for the following variables: age, sex, hepatitis status and related indicators, total bilirubin levels, albumin concentrations, TSUV_max_, LSUV_mean_, blood cell counts, and tumor characteristics (including the number of tumors, tumor diameter, degree of differentiation, and stage).

### 2.3. Follow-Up

Follow-up evaluations were conducted quarterly by telephone during the first year after surgery and semi-annually thereafter. These assessments included chest and abdominal CT scans, liver MRI, and AFP quantification. The observation period spanned from the date of surgery to the date of last follow-up or death, whichever occurred first. Survival time was defined as the interval from surgery to either the date of death or the final follow-up.

### 2.4. Data Analysis

Data processing was performed using SPSS 25.0 statistical software. For the comparison of two groups, Student’s *t*-tests were performed for continuous variables, whereas chi-square tests were conducted for categorical variables. Analysis of variance was used to assess differences in mean values between more than two groups. Associations between variables were assessed using Spearman’s rank correlation analysis. To determine the optimal cut-off values of TLR and NLR for predicting postoperative survival, receiver operating characteristic (ROC) curve analyses were performed. Multicollinearity was evaluated using the variance inflation factor (VIF). Univariate and multivariate Cox proportional hazards regression models were employed to identify independent prognostic factors for overall survival (OS) and disease-free survival (DFS). Survival differences between groups were compared using Kaplan–Meier survival curves and the log-rank test, and survival rates were subsequently estimated. Statistical significance was set at *p* < 0.05.

## 3. Results

### 3.1. Baseline Characteristics of Patients

Eighty-nine patients were ultimately included in the statistical analysis. The mean age was 57.4 ± 11.93 years (range: 24–77 years), and 71 patients (79.8%) were male. The most common underlying disease was simple infection with hepatitis B virus (75/89; 84.3%), followed by simple infection with hepatitis C virus (4/89; 4.5%), hepatitis B and C virus co-infection (4/89; 4.5%), alcohol-related infection (3/89; 3.4%), and other diseases (3/89; 3.4%). Among all 89 patients, 55 (61.8%) had concomitant liver cirrhosis, and 14 (15.7%) had concomitant metabolic dysfunction-associated steatotic liver disease (MASLD). Among all the MASLD patients, there was no active hepatitis. Only the hepatitis B core antibody showed a slight increase.

The ALBI score, which is less subjective than other common scoring systems, was used to evaluate preoperative liver function. The average ALBI score of all patients was −2.81 ± 0.46, with 66 patients (74.2%) classified as Grade 1, 19 patients (21.3%) as Grade 2, and four patients (4.5%) as Grade 3. The average preoperative AFP concentration was 2035.98 ± 8558.89 ng/mL. The average maximum tumor diameter was 5.71 ± 3.54 cm (range: 0.8–17 cm), and the average number of tumors was 1.24 ± 0.60. The average preoperative SUV_max_, measured using PET/CT, was 6.04 ± 3.08 (range: 0.87–15.71), and the average SUV_mean_ was 2.42 ± 0.47 (range: 1.5–3.9). The average absolute neutrophil count was 3.19 ± 1.14 (range: 1.1–6.6), and the average absolute lymphocyte count was 1.59 ± 0.61 (range: 0.4–3.7).

Tumor stages were classified according to the China Liver Cancer staging system. Forty one patients (46.1%) had stage IA, 38 (42.7%) had stage IB, seven (7.9%) had stage IIA, and three (3.4%) had stage IIB tumors. The degree of tumor differentiation was classified as well-differentiated (23/89; 25.8%), moderately well-differentiated (6/89; 6.7%), moderately differentiated (44/89; 49.4%), moderately poorly differentiated (12/89; 13.5%), and poorly differentiated (2/89; 2.2%). The degree of differentiation was unclear in two (2.2%) cases. The Edmondson–Steiner grade of the tumor was as follows: Grade 1 (24/89; 26.9%), Grade 2 (50/89; 56.2%), and Grade 3 (15/89; 16.9%). Detailed baseline characteristics are presented in Table 1.

### 3.2. Analysis of Correlations Between TLR, NLR, and Tumor Differentiation

A significant correlation was observed between the SUV_max_ and NLR (r = 0.210, *p* = 0.048), whereas no significant correlation was found between the TLR and NLR (r = 0.151, *p* = 0.158). However, the TLR was significantly positively correlated with the degree of HCC differentiation (r = 0.348, *p* < 0.01), which was stronger than the relationship observed for SUV_max_ (r = 0.291, *p* < 0.01). The one-way analysis of variance revealed significant differences (*p* < 0.01) among at least one group of TLRs with different Edmondson–Steiner grades. Further post hoc tests indicated that there were significant differences between Grade I and Grade II HCC (*p* = 0.02, 95% CI, −1.51–0.98), as well as between Grade I and Grade III HCC (*p* < 0.01, 95% CI, −2.26–0.64). Compared with that of Grade I disease, Grade II and Grade III HCC were associated with higher TLR values; however, there was no significant difference between Grade II and Grade III disease (*p* = 0.256, 95% CI, −1.59–0.29).

### 3.3. ROC Curve Analysis

The area under the ROC curve for the TLR was 0.654, with a cut-off value of 2.19 (sensitivity, 67.9%; specificity, 59%; *p* = 0.020). The area under the ROC curve for the NLR was 0.65, with a cut-off value of 2.29 (sensitivity, 60.7%; specificity, 70.5%; *p* = 0.024).

### 3.4. Cox Regression Analysis and Post-Surgical Survival Curves of OS and DFS

In the univariate Cox regression analysis, an AFP concentration > 100 ng/mL, a TLR > 2.193, and an NLR > 2.29 were significantly associated with OS and DFS in patients with HCC. The ALBI score was only significantly associated with OS. All variables intended for inclusion in the Cox model underwent variance inflation factor (VIF) analysis, which showed that all VIF values were below 1.5. In the multivariate analysis, a TLR > 2.19, an NLR > 2.29, and an AFP concentration > 100 ng/mL were independently associated with both OS and DFS in patients with HCC after liver resection. Similar to what was observed in the univariate analysis, the ALBI score was only independently associated with OS. Details are presented in Table 2.

**Figure 3:**
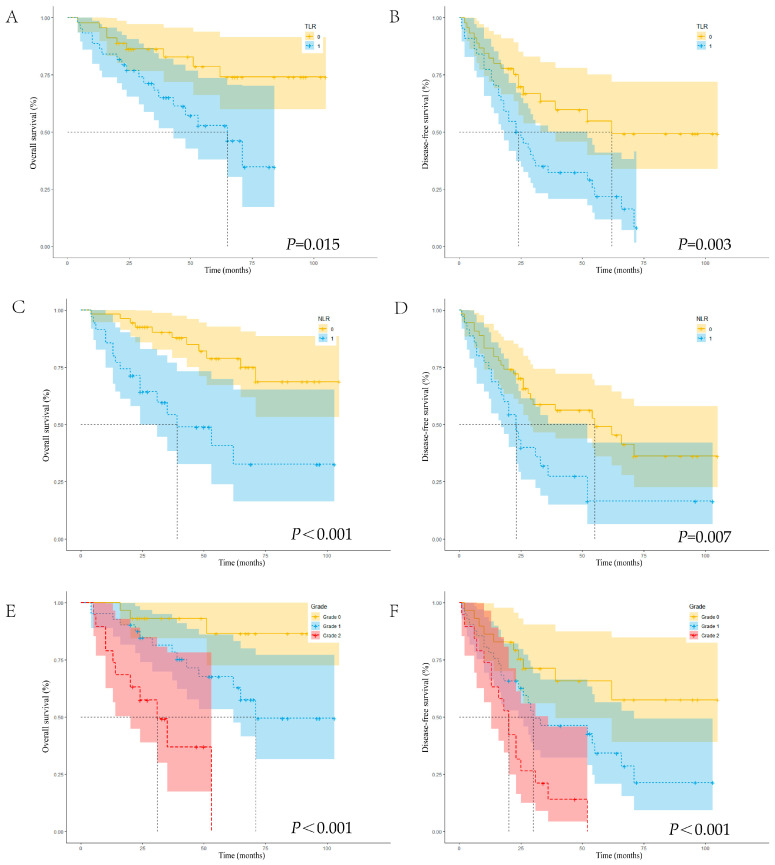
Kaplan–Meier survival curves for OS and DFS in patients with HCC. (**A**) OS rates stratified by the TLR; (**B**) DFS rates stratified by the TLR; (**C**) OS rates stratified by the NLR; (**D**) DFS rates stratified by the NLR; (**E**) OS rates stratified by the TLR–NLR grade; (**F**) DFS rates stratified by the TLR–NLR grade. OS, overall survival; DFS, disease-free survival; HCC, hepatocellular carcinoma; TLR, tumor-to-liver ratio; NLR, neutrophil–lymphocyte ratio.

Of the 89 patients included in the study, 28 died and 52 experienced relapse during the follow-up period. Among the 89 patients, three were lost to follow-up, and the follow-up rate was 96.6%. No patients underwent liver transplantation after the surgery. Among the 28 deaths that occurred, 27 were predominantly attributed to HCC progression, including tumor burden-induced liver failure. One patient died of heart disease after tumor recurrence. The median follow-up time for the entire cohort was 53 months (95% CI, 44.869–61.131 months), calculated using the reverse Kaplan–Meier method. The Kaplan–Meier survival analysis revealed cumulative survival rates of 91.0%, 77.0%, and 63.1% at 1, 3, and 5 years, respectively, and the DFS rates were 79.8%, 46.9%, and 38.5%, respectively. Compared with that of the high-TLR group, the low-TLR group exhibited better 1-, 3-, and 5-year OS (95.6%, 82.8%, and 74.1% vs. 86.4%, 68.2%, and 46.2%, respectively; *p* = 0.015) (Figure 3A) and DFS (82.2%, 63.4%, and 49.3% vs. 72.7%, 32.3%, and 21.8%, respectively; *p* < 0.01) (Figure 3B), suggesting that a high TLR was associated with a poor prognosis. Compared with that of the high-NLR group, the low-NLR group also exhibited better 1-, 3-, and 5-year OS (96.3%, 87.9%, and 74.9% vs. 80.0%, 54.3%, and 32.6%, respectively; *p* < 0.01) (Figure 3C) and DFS (83.3%, 56.1%, and 45.3% vs. 74.3%, 27.3%, and 16.4%, respectively; *p* < 0.01) (Figure 3D), indicating that a high NLR was associated with a poor prognosis.

### 3.5. Assessment of the Performance of the Novel Scoring System for Predicting OS Before Surgery in Patients with HCC

Based on the relationship between the TLR and NLR and the previously identified cut-off values, a novel scoring system was established to predict the prognosis of patients with HCC following liver resection. Depending on whether the values did or did not exceed the established cut-offs, 1 or 0 points were assigned for each parameter, and the patients were classified into three subgroups. More specifically, the group with a TLR > 2.19 and an NLR > 2.29 was assigned 2 points; the group with a TLR ≤ 2.19 and an NLR > 2.29 or a TLR > 2.19 and an NLR ≤ 2.29 was assigned 1 point; and the group with a TLR ≤ 2.19 and an NLR ≤ 2.29 was assigned 0 points. There were 29 patients in the 0-point group, three (10.3%) of whom died and 10 (34.5%) of whom experienced disease recurrence. Forty-one patients comprised the 1-point group, 14 (34.1%) of whom experienced death and 25 of whom exhibited recurrence (61.0%). The 2-point group was composed of 19 patients, 11 (57.9%) of whom died and 17 (89.5%) of whom experienced recurrence.

Further KM curves were subsequently plotted to continue the survival analysis (Figure 3E,F). The 1-year, 3-year, and 5-year OS rates of the 0-point group were 96.6%, 93.1%, and 86.5%, respectively; in the 1-point group, they were 92.7%, 78.3%, and 62.8%, respectively, and in the 2-point group, they were 73.7%, 36.9%, and 0%, respectively. There were significant intergroup differences (*p* < 0.01), and the OS of the 0-point group was significantly longer than that of the 1-point and 2-point groups. The DFS rates were 86.2%, 65.7%, and 57.5% for the same time points, respectively, in the 0-point group; in the 1-point group, they were 78.0%, 46.2%, and 28.4%, respectively, and in the 2-point group, they were 63.2%, 14.0%, and 0%, respectively. There were also significant differences in DFS between the groups (*p* < 0.01).

**Figure 4:**
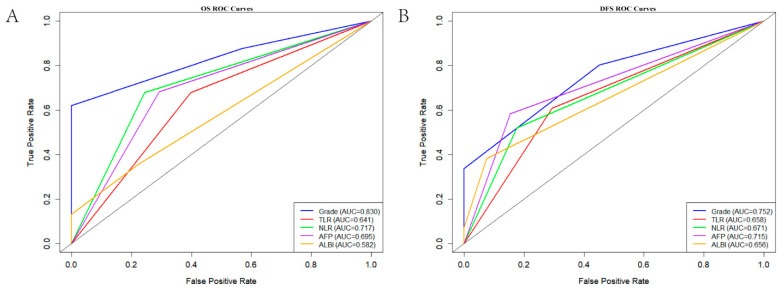
Comparison of the AUC values of the ROC curves for the TLR–NLR grade, TLR, NLR, AFP level, and ALBI grade in predicting 5-year OS (**A**) and DFS; (**B**) of patients with HCC. AUC, area under the curve; ROC, receiver operating characteristic; TLR, tumor-to-liver ratio; NLR, neutrophil–lymphocyte ratio; AFP, alpha-fetoprotein; ALBI, Albumin–bilirubin grade; OS, overall survival; DFS, disease-free survival.

To evaluate the scoring system’s predictive value in terms of survival outcomes in patients with HCC, internal validation was conducted, and a time-dependent ROC curve model was established to predict the 5-year OS and DFS of patients (Figure 4). The area under the ROC curve values of the joint TLR–NLR scoring system were 0.830 and 0.752 for 5-year OS and DFS, respectively, both of which were superior to those of the scoring systems based on single indicators.

## 4. Discussion

Surgery has long been the most important treatment for HCC; however, the high postsurgical recurrence rate remains a major obstacle that negatively impacts the long-term survival of patients and poses a major challenge for surgeons. Therefore, developing accurate techniques for preoperative prognostic prediction in HCC is crucial for identifying optimal surgical candidates. Early diagnosis followed by surgical intervention has been associated with 5-year survival rates as high as 70% [17]. Consistent with these findings, our study observed 3-year and 5-year survival rates of 78.7% and 63.1%, respectively, with the majority (88.8%) of patients presenting at stage Ia or Ib. According to the China Liver Cancer Staging criteria, patients with stage Ia and Ib HCC can achieve post-treatment 5-year survival rates of up to 77.4% and 53.2%, respectively [18], respectively; this is consistent with the present findings and, to some extent, indicates that the diagnosis and treatment in this study population conformed to the China Liver Cancer Staging standards.

Multiple studies have investigated factors influencing long-term survival in patients with HCC. For instance, although AFP is widely used in the diagnosis and prognostic evaluation of HCC, it is elevated in only approximately 60% of cases. From a prognostic standpoint, AFP-positive patients exhibit a lower 5-year survival rate (26.7%) compared to those who are AFP-negative (56.5%) [19]. Consistent with these findings, our analysis identified elevated AFP levels (>100 ng/mL) as an independent risk factor for both OS and DFS in patients undergoing HCC resection. Several liver function scoring systems are currently used in clinical practice, including the Child-Pugh, Model for End-Stage Liver Disease (MELD), and ALBI scores. The ALBI score described in the 2022 version of the Barcelona Clinic Liver Cancer staging system has been identified as a prognostic predictor in patients with HCC [20]. The Child-Pugh score considers ascites and hepatic encephalopathy factors, the characterization of which can be somewhat subjective, whereas the MELD score is predominantly used for patients with end-stage liver cirrhosis, and there were no patients meeting that description among this cohort. In contrast, ALBI scoring is simple, easy to perform, completely objective, and provides better discriminability [21]; therefore, in the present study, the ALBI score was selected to evaluate the liver function of patients before surgery, and it was identified as an independent postsurgical prognostic factor for OS in the multivariate analysis. However, there was no significant difference between patients with ALBI Grades of 2 and 1, possibly owing to the fact that the proportion of patients with early-stage HCC was relatively high and OS was relatively long in this cohort. For such patients, the predictive ability of the ALBI score may have certain limitations. It is also important to note that ALBI score was not significantly associated with DFS. Furthermore, there was no significant difference in tumor recurrence between patients with different ALBI grades.

SUV_max_, one of the most important parameters derived from PET, can accurately assess the metabolic status of tumors and is commonly used for evaluating patient prognosis. SUV_max_ and the indicators derived from it (TSUV_max_/LSUV_max_ and TSUV_max_/LSUV_mean_) have been proven to be accurate predictors of some biological characteristics of tumors that are known to be associated with a poor prognosis in patients with HCC, such as poor tumor differentiation, microsatellite foci, and microvascular invasion [22,23,24,25], which is consistent with the present observations. However, few studies have investigated the correlation between the TLR and a poor prognosis following surgical resection for the treatment of HCC. One complication of relying on SUV_max_ as a prognostic indicator is the fact that basic characteristics of patients undergoing treatment in different centers can vary, leading to discrepancies in the SUV_max_ cut-off values, and no unified standards have been established to date. In addition, compared to SUV_max_, SUV ratios exhibit greater predictive value in prognostic assessments, and TSUV_max_/LSUV_mean_ has been shown to outperform TSUV_max_/LSUV_max_ [26]. Previous studies have shown that patients with a TLR exceeding a cut-off value of 2 were more likely to experience a poor prognosis, whereas those with a TLR < 2 were more likely to have better survival outcomes [27]. In this study, the cut-off value of the TLR was set at 2.19, which was identified as an independent prognostic factor for OS and DFS post-surgery in patients with HCC, and there were significant differences in OS and DFS between the two groups stratified by this value. Furthermore, the TLR was also confirmed to be significantly and positively correlated with HCC differentiation (r = 0.348, *p* < 0.01), and the correlation was stronger than that observed for SUV_max_. Our research also suggested that a higher TLR value may be indicative of a more severe Edmondson–Steiner grade.

Chronic inflammation and tumor development have been shown to be intrinsically linked, with the relationship serving as a contributing factor to tissue malignancy, new blood vessel formation, tumor progression, metastasis, and recurrence, and drug resistance [28,29,30], providing an immune microenvironment conducive to tumor growth. These effects are more prominent in patients with HCC than in those with other cancer types. Of the 89 patients included in the study, 83 (93.2%) were infected with hepatitis B or C virus. Inflammation-related indicators have been widely used to predict the prognosis of patients with HCC, including C-reactive protein levels, the NLR, the platelet-to-lymphocyte ratio, and the lymphocyte-to-monocyte ratio, among others. A study published in *Nature* suggested that tumor-associated neutrophils exert a pro-tumor effect in HCC, and their depletion can slow tumor progression [29]. Previous studies have also reported a significant association between a high NLR and poor prognosis in patients with HCC who have undergone liver resection [31]. In the present study, using 2.29 as the NLR cut-off value yielded significant differences in both OS and DFS between the two groups of patients. Moreover, among all of the prognostic factors ultimately included in the analysis, the NLR exhibited the greatest statistical significance.

Single predictive indicators exhibit only modest predictive value, and they are often unsuitable for distinguishing between the characteristics of different patients. Therefore, this study established a novel model in which the PET/CT-derived TLR, which reflects the metabolic characteristics of the tumor itself, was combined with the NLR, which reflects the immune microenvironment of the host, to comprehensively evaluate the prognosis of patients with HCC who had undergone liver resection. The proliferation of tumor cells and abnormal vascularization accelerate oxygen consumption, leading to tumor hypoxia, which manifests as increased FDG uptake and accelerated metabolism [32], potentially inducing a nonspecific inflammatory response in the host, which is consistent with the significant correlation observed between the SUV_max_ and NLR in the present study (r = 0.210, *p* = 0.048). Similar conclusions have been reported in other studies that have investigated primary tumors [33]. This phenomenon may be explained by inflammation-associated pathways. First, inflammation can induce angiogenesis through hypoxia and vascular endothelial growth factor secretion within the tumor microenvironment, and the resulting neovascularization contributes to increased FDG uptake [34]. Second, infiltrating inflammatory cells, such as neutrophils and macrophages, exhibit high FDG avidity, thereby further enhancing the metabolic signal across the tumor [35]. Combinations of SUV-related and inflammatory indicators have been effectively employed in the prognostic assessment of non-small cell lung cancer as well as colon, breast, and pancreatic cancers, among other malignant tumors. Multivariate Cox regression analysis demonstrated that a NLR > 2.29 and a TLR > 2.19 are independent predictors of both OS and DFS in the cohort of patients with hepatocellular carcinoma (HCC) who have undergone liver resection. Based on these findings, a joint TLR–NLR scoring system was established to categorize patients into three subgroups for further assessment of the postoperative prognosis. Internal validation was conducted to prove that the system had predictive value for assessing the prognosis of patients with HCC. Patients with a score of 0 (TLR ≤ 2.19 and NLR ≤ 2.29) exhibited the longest OS and DFS, whereas those with a score of 2 (TLR > 2.19 and NLR > 2.29) experienced significantly shorter OS and DFS compared to that of the former group.

Historically, the characteristics used to identify patients with a high risk of HCC recurrence have been predominantly based on the biological features of tumors, including their number and diameter, the degree of pathological microvascular invasion, the presence of microsatellite foci, and the Edmondson–Steiner grade [36]. A considerable number of patients with a suspected high risk of recurrence are often identified based on the assessment of postoperative pathological conditions. The present study confirmed that the scoring system was capable of accurately identifying those with a high recurrence risk preoperatively based on a combination of the TLR and NLR, which could be useful for facilitating the selection of treatment regimens. For patients with a preoperative score of 2, it may be necessary to evaluate the possibility of using neoadjuvant or conversion therapy before surgery to achieve a better prognosis. Simultaneously, patients should be closely monitored after surgery and could benefit from the administration of further relevant systemic or local treatments.

This study has certain limitations. First, this was a single-center retrospective analysis with a relatively limited sample size. Since PET/CT is not a mandatory clinical examination before liver resection and its cost was relatively high, the number of patients who meet the criteria and receive this examination early was also limited. Moreover, the majority of patients comprising this cohort had hepatitis virus-related conditions, whereas relatively few patients had MASLD which resulted in a relatively low incidence of liver cirrhosis. In fact, the incidence of liver cirrhosis may have a certain impact on overall survival. Consequently, the factors that were identified as statistically significant in the present study should be validated in future prospective studies with larger and more diverse cohorts, including populations diagnosed with MASLD. Second, the inclusion of a higher proportion of patients with early- and mid-stage HCC resulted in a relatively prolonged OS time; furthermore, the follow-up period was relatively short, and the results may not be applicable to patients with advanced HCC.

## 5. Conclusions

Ultimately, the preoperative TLR and NLR were identified as independent prognostic factors for OS and DFS in patients with early to intermediate-stage HCC undergoing hepatectomy. The proposed scoring system, which incorporates both of these factors, simultaneously reflects the metabolic characteristics of the tumor and the immune microenvironment of the host, enabling more accurate patient classification and better prognostic evaluation.

## Figures and Tables

**Table 1:** Demographic and Clinical Characteristics of the Participants.

Characteristics	*n* (%) or Mean ± SD
Age (years), mean	57.40 ± 11.93
Sex (male), *n* (%)	71 (79.8)
Underlying disease, *n* (%)	
Hepatitis B	75 (84.3)
Hepatitis C	4 (4.5)
Hepatitis B and C	4 (4.5)
Alcohol-related infection	3 (3.4)
Other	3 (3.4)
Cirrhosis	55 (61.8)
MASLD	14 (15.7)
Lymphocyte count (10^9^/L), mean	1.59 ± 0.61
Platelet count (10^9^/L), mean	163.55 ± 64.18
Neutrophil count (10^9^/L), mean	3.19 ± 1.14
Total bilirubin (μmol/L), mean	15.31 ± 11.32
Albumin (g/L), mean	41.56 ± 4.85
ALBI grade, mean	−2.81 ± 0.46
TSUV_max_, mean	6.04 ± 3.08
LSUV_mean_, mean	2.42 ± 0.47
AFP (ng/mL), mean	2034.98 ± 8558.89
Tumor size (cm), mean	5.71 ± 3.54
Number of tumors, mean	1.24 ± 0.60
Barcelona Clinic Liver Cancer stage, *n* (%)	
A	79 (88.8)
B	10 (11.2)
China Liver Cancer stage, *n* (%)	
IA	41 (46.1)
IB	38 (42.7)
IIA	7 (7.9)
IIB	3 (3.4)
Edmondson–Steiner grade, *n* (%)	
Grade I	24 (26.9)
Grade II	50 (56.2)
Grade III	15 (16.9)

Abbreviations: SD, standard deviation; ALBI, Albumin–bilirubin; TSUV_max_, maximum standardized uptake value of tumor tissue; LSUV_mean_, average standardized uptake value of normal liver tissue; AFP, alpha-fetoprotein.

**Table 2:** Prognostic Factors for OS and DFS.

Variables	OS				DFS			
	Univariate HR (95% CI)	*p*	Multivariate HR (95% CI)	*p*	Univariate HR (95% CI)	*p*	Multivariate HR (95% CI)	*p*
Age (≥60 years)	1.981 (0.926–4.239)	0.078			1.562 (0.902–2.707)	0.112		
Sex (male)	0.568 (0.250–1.292)	0.177			0.475 (0.259–0.871)	0.016	0.647 (0.333–1.258)	0.199
ALBI grade		0.031		0.020		0.341		
Grade 1	Reference		Reference		Reference			
Grade 2	0.942 (0.376–2.361)	0.899	0.552 (0.209–1.458)	0.231	1.187 (0.627–2.249)	0.598		
Grade 3	5.063 (1.470–17.442)	0.010	4.689 (1.268–17.339)	0.021	2.680 (0.808–8.890)	0.107		
Viral hepatitis	0.43 (0.129–1.434)	0.170			0.642 (0.229–1.795)	0.398		
Cirrhosis	1.208 (0.546–2.676)	0.641			1.133 (0.642–1.997)	0.667		
MASLD	1.209 (0.490–2.983)	0.680			1.198 (0.615–2.334)	0.596		
NLR (>2.29)	3.896 (1.802–8.420)	0.001	4.800 (2.045–11.263)	<0.001	2.101 (1.206–3.658)	0.009	2.115 (1.155–3.875)	0.015
TLR (>2.19)	2.592 (1.167–5.760)	0.019	2.946 (1.281–6.774)	0.011	2.337 (1.315–4.152)	0.004	2.061 (1.106–3.842)	0.023
AFP (>100 ng/mL)	3.103 (1.428–6.739)	0.004	2.515 (1.125–5.623)	0.025	2.700 (1.544–4.719)	<0.001	2.031 (1.096–3.766)	0.024
Number of tumors	1.081 (0.571–2.049)	0.811			1.046 (0.665–1.644)	0.847		
Tumor size (>5 cm)	1.548 (0.728–3.289)	0.256			1.798 (1.020–3.171)	0.043	1.046 (0.551–1.987)	0.890
Differentiation	1.321 (0.921–1.894)	0.130			1.054 (0.818–1.358)	0.686		

Abbreviations: OS, overall survival; DFS, disease-free survival; HR, hazard ratio; CI, confidence interval; ALBI, Albumin–bilirubin; MASLD, metabolic dysfunction-associated steatotic liver disease; NLR, neutrophil–lymphocyte ratio; TLR, tumor-to-liver ratio (maximum standardized uptake value in tumor tissue/mean standardized uptake value of normal liver tissue); AFP, alpha-fetoprotein. Variables with *p* < 0.05 in the univariate analysis were retained in the multivariate analysis.

## Data Availability

The raw data supporting the conclusions of this article are available from the corresponding author without reservation.

## Abbreviations

The following abbreviations are used in this manuscript:

AFP	Alpha fetoprotein
ALBI	Albumin–bilirubin
AUC	Area under the curve
CI	Confidence interval
CT	Computed tomography
DFS	Disease-free survival
HCC	Hepatocellular carcinoma
HR	Hazard ratio
LSUV_mean_	Mean standardized uptake value of liver tissue
MASLD	Metabolic dysfunction-associated steatotic liver disease
MELD	Model for End-stage Liver Disease
NLR	Neutrophil–lymphocyte ratio
OS	Overall survival
PET	Positron emission tomography
ROC	Receiver operating characteristic
SUV	Standardized uptake value
SUV_max_	Maximum standardized uptake value
SUV_mean_	Mean standardized uptake value
TLR	Tumor-to-liver ratio
TSUV_max_	Maximum standardized uptake value of tumor tissue
VIF	Variance inflation factor
